# Expression of pY397-FAK and Its miR Regulators Drive Dedifferentiation in the Thyroid Neoplasia Spectrum

**DOI:** 10.3390/cells12131721

**Published:** 2023-06-26

**Authors:** Valentina Ignjatović Jocić, Jelena Janković Miljuš, Tijana Išić Denčić, Vladan Živaljević, Svetislav Tatić, Ilona Đorić, Sonja Šelemetjev

**Affiliations:** 1 Department of Endocrinology and Radioimmunology, Institute for the Application of Nuclear Energy—INEP, University of Belgrade, Banatska 31b, 11000 Belgrade, Serbia; 2 Center for Endocrine Surgery, University Clinical Center of Serbia, Doktora Subotića 13, 11000 Belgrade, Serbia; 3 Institute for Pathology, Faculty of Medicine, University of Belgrade, Doktora Subotića Starijeg 1, 11000 Belgrade, Serbia

**Keywords:** pY397-FAK, miRs, thyroid carcinoma, neoplastic dedifferentiation, differential diagnostics

## Abstract

Thyroid carcinomas are growing malignancies worldwide. They encompass several diagnostic categories with varying degrees of dedifferentiation. Focal adhesion kinase is involved in cellular communication and locomotion. It is regulated on a posttranscriptional level by miR-7, miR-135a, and miR-138 and on a posttranslational level by autophosphorylation at Y397 (pY397-FAK). We related regulators of FAK with histologic dedifferentiation, clinicopathological factors, and differential diagnosis in the thyroid neoplasia spectrum. We classified 82 cases into 5 groups with increasing aggressiveness: healthy tissue, follicular and classical variants of papillary thyroid carcinoma (PTC), dedifferentiated PTC, and anaplastic carcinoma. MiRs were analyzed by RT-qPCR. Protein expression of pY397-FAK was analyzed by immunohistochemistry (separately in the membrane, cytoplasm, and nuclear compartment) and Western blot. All three miRs were upregulated in healthy tissue compared to malignant, while pY397-FAK was downregulated. MiRs and pY397-FAK were not mutually correlated. MiR-135a-5p was decreasing while membranous and cytoplasmic pY397-FAK increased with dedifferentiation. Neither miR correlated with clinicopathological factors. MiR-135a-5p, miR-138-5p, and membranous and cytoplasmic pY397-FAK discriminated the follicular from the classical variant of PTC. Disturbances of FAK regulation on different levels contribute to neoplastic dedifferentiation. pY397-FAK exerts its oncogenic role in the membrane and cytoplasm. Diagnostically, miRs-135a-5p, miR-138-5p, and membranous and cytoplasmic pY397-FAK differentiated between classical and follicular PTC.

## 1. Introduction

Thyroid carcinomas are the most frequently occurring neoplasia of the endocrine system and are among the most rapidly growing malignancies worldwide [1]. They encompass a number of diseases with different genetic signatures, biological behavior, and, consequently, clinical manifestations. The vast majority of diagnosed cases belong to well-differentiated papillary thyroid carcinomas (PTC) characterized by high survival rates and good responses to common treatment methods. On the opposite pole of the spectrum, anaplastic carcinomas (ATC) are associated with poor prognosis and a median survival time of less than 6 months regardless of stage [2], although a recent study shows prolonged survival with the use of vemurafenib in *BRAF*(V600E)-mutated anaplastic thyroid cancer [3] In between, there is a heterogeneous group of rare papillary carcinomas going through different degrees of differentiation and morphological features. As the early stages of these conditions histologically often resemble each other, much effort has been put into finding molecular markers for thyroid nodule differential diagnostic and prognostic stratification in order to tailor treatment according to each patient’s requirements. Although several markers have found their way to clinical practice, their reliability is still in question. Hence, the field is still much in need of ancillary or alternative candidates.

Focal adhesion kinase (FAK) is a 125 kDa non-receptor tyrosine kinase that was initially shown to be located at focal adhesions, i.e., contact points for the cell with the extracellular matrix regulating its communication with the surroundings. In response to external cues, FAK triggers signaling cascades in a variety of cellular activities, thereby playing a vital role in cellular communication and locomotion. It receives extracellular signals through transmembrane receptors such as integrins, cytokines, and growth factors, thereby integrating biochemical signals and mechanical forces to control cell motility. FAK is overexpressed in cancer cells and takes part in the advancement of tumors to a malignant phenotype. Increased mRNA levels, protein levels, and activation of FAK are related to cancer metastatic and invasive properties [4,5]. Given its function, it was initially thought that FAK exerts its function in the membrane and cytosol. Subsequently, however, a nuclear function of FAK has been revealed. Upon translocation to the nucleus, FAK regulates gene expression by binding transcription factors such as p53 and promotes malignant cell proliferation and survival [6,7]. For example, it can promote the degradation of p53 protein by ubiquitination, hence directly inducing proliferative activity, or control GATA4 and IL-33 expression and consequently reduce inflammatory responses and immune escape [4]. This implies disparate mechanisms of oncogenic action of FAK depending on subcellular localization. Several studies identified overexpression of FAK in thyroid tumors including papillary, follicular, and anaplastic [8,9,10]. However, in order to exert some of its biological functions, FAK needs to go through activation by autophosphorylation of tyrosine residues. It has at least six tyrosine phosphorylation sites including −407, −576, −577, −925, and −397 [11] Among these, phosphorylation at Y397 is considered the most important event as it enables FAK to kick off numerous downstream pathways through its interaction with Src family kinases phosphatidylinositol-3 kinase, growth factor receptor bound protein 7, and additional signaling molecules in response to integrin adherence to various extracellular matrix adhesive molecules [12]. Hence, the assessment of FAK phosphorylation at Y-397 is considered an indirect estimation of its activity. In our previous research, we have demonstrated an overexpression of posttranslational modification of FAK phosphorylated at position 397 (pY397-FAK) in relation to unfavorable clinicopathological factors [13]. However, its utility as a marker for differential diagnosis or neoplastic dedifferentiation has not been reported so far.

Apart from posttranslational regulation, FAK is also regulated on the posttranscriptional level. Among the main regulators of *FAK* gene expression are micro RNAs (miRs), a class of small non-coding RNAs known to interact with mRNAs through complementary base-pairing and influence their stability. In humans, it is estimated that over 2500 miRs regulate over 60% of human genes and take part in every aspect of cellular activity [14]. Disturbances of miR expression, along with mutations in oncogenes tumor/suppressor genes and genes that provide genome stability contribute to many pathological disorders including proliferative diseases. Three miRs have been shown to target and silence *FAK* in carcinomas other than thyroid: miR-7 [15,16] miR-135a, and miR-138 [17,18]. Direct interaction of these miRs with FAK 3′ UTR was shown in breast cancer cell lines (miR-7), resulting in inhibition of epithelial-to-mesenchymal transition and cancer metastasis [15], and in various cancer cell lines (miR-135a and miR-138), showing induced inhibition of cell invasion and increased sensitivity to chemotherapy [17]. In general, these miRs are downregulated in neoplastic tissues compared to their healthy counterparts. They are dominantly tumor suppressor factors inhibiting proliferation, migration, and survival and promoting apoptosis. This has been demonstrated in a number of malignancies including thyroid [19,20,21]. However, for miR-135a and -138, oncogenic functions were also reported. For example, miR-135 is upregulated in bladder, oral, colorectal, and liver cancers, while it exerts dual roles in breast, gastric, lung, and pancreatic cancers and head and neck squamous cell carcinoma [22].

Given all of the above, this study aimed to elucidate the role of FAK miR regulators (miR-7-5p, -135a-5p, and -138-5p) in thyroid oncogenesis and investigate their relation to pY397-FAK expression. Beyond this scope, we intended to examine whether the expression of these miRs and the localization of pY397-FAK in each subcellular compartment (membrane, cytoplasm, or nucleus) can be a hallmark of gradual malignancy dedifferentiation towards more lethal forms. Finally, we attempted to resolve a common diagnostic dilemma in thyroid pathology, i.e., investigate whether the expression of the previously mentioned molecules could be of use in differentiating the most common classes of papillary carcinoma: the follicular histologic variant (PTC-fv) as a more indolent form and the classic histologic variant (PTC-clv), tending to behave more aggressively.

## 2. Materials and Methods

### 2.1. Tissue Samples and Clinical Data

The study was conducted according to the Declaration of Helsinki and approved by the Ethics Committee of the Clinical Centre of Serbia (reference no.: 140/15), Belgrade, Serbia. All patients involved in the study signed informed consent forms for using their biological material for research purposes. Thyroid tissue specimens were obtained from patients who underwent thyroidectomy at the Clinic for Endocrine Surgery, University Clinical Centre of Serbia. Tissue samples, derived from removed thyroid tumors, were snap-frozen in liquid nitrogen and stored at −80 °C for RNA and protein isolation. Along with tumor tissue, conditionally healthy tissue was sampled from a distant location, possibly from the contralateral lobe. Additionally, tissue samples from the same patients were fixed in formalin and embedded in paraffin, and then used for immunohistochemical staining. Histopathologic diagnosis was confirmed by the pathologist following the World Health Organization classification of thyroid tumors [23]. Cases were stratified and scored into four groups with increasing histotype aggressiveness and dedifferentiation: follicular variant (grade 1), classical variant (grade 2), rare types of PTC (tall cell, columnar cell, diffuse sclerosing, cribriform-morular, solid, hobnail, and Warthin-like variants; grade 3) and anaplastic thyroid carcinoma (grade 4). Patients were staged according to the TNM staging system in accordance with the American Joint Committee on Cancer (AJCC) [24]. Personal and clinical data of the patients, including age, gender, tumor size, presence of lymph node metastasis (LNM), intraglandular dissemination (ID), degree of tumor infiltration (DI), extra-thyroid invasion (EI), pT status, and TNM stage were obtained from pathology reports. The degree of tumor infiltration (DI) was evaluated according to Basolo et al. [25] as follows: DI-1, totally encapsulated tumors; DI-2, non-encapsulated tumors without thyroid capsule invasion; DI-3, tumors with thyroid capsule invasion; and DI-4, tumors with extra-thyroid invasion. In order to simplify data analysis and make data interpretation more straightforward, DI, pT, and TNM categories were grouped as Low (including stages 1 and 2) and High (including stages 3 and 4).

### 2.2. Total RNA Extraction, Reverse Transcription (RT), and Quantitative PCR (qPCR)

Total RNA extraction from 82 tissue samples was performed using TRIzol reagent (Ambion, Carlsbad, CA, USA) following the manufacturer’s instructions. The concentration and purity of extracted RNA were determined by an Epoch microplate spectrophotometer (BioTek, Winooski, VT, USA). Before cDNA synthesis, a total of 400 ng of RNA was treated with DNase 1 Grade (Sigma-Aldrich, Burlington, MA, USA) according to the protocol recommended by the manufacturer. DNase-treated total RNA was reverse transcribed using 100 U of RevertAID reverse transcriptase, 0.5 mM dNTPs, and miR-specific stem-loop RT primers (250 nM each) in the final volume of 20 µL. The running method included sequential incubation at 16 °C for 30 min, 42 °C for 30 min, and 85 °C for 5 min. Reversibly transcribed RNA was then quantified by qPCR using an SYBR Green PCR Master Mix (Applied Biosystems, Foster City, CA, USA) following the manufacturer’s instructions. QPCR was performed on a 7500 Real-Time PCR System (Applied Biosystems, Foster City, CA, USA) with the following conditions: hold stage at 50 °C for 2 min and at 95 °C for 10 min, followed by 40 cycles of denaturation at 95 °C for 15 s, and annealing/extension at 60 °C for 60 s. For each miR, the PCR reaction was performed in triplicate. Ct values were normalized to U6 small RNA Ct values which was an internal control. The miR’s relative expression level was determined by the 2^−ΔCt^ method. In order to reduce skewness and achieve normal distribution, all data were transformed logarithmically. An inter-run calibrator was used to control for the differences among different qPCR runs (a cDNA ran across all plates).

Stem-loop RT and qPCR primers were designed as follows: stem-loop miR-7-5p GTCGTATCCAGTGCAGGGTCCGAGGTATTCGCACTGGATACGACAACAACAA, Stem-loop miR-135a-5p GTCGTATCCAGTGCAGGGTCCGAGGTATTCGCACTGGATACGACTCACATA, Stem-loop miR-138-5p GTCGTATCCAGTGCAGGGTCCGAGGTATTCGCACTGGATACGACCGGCCT, Stem-loop U6 GTCGTATCCAGTGCAGGGTCCGAGGTATTCGCACTGGATACGACAAAAATATGG, Forward miR-7-5p CGGCGGTTGGAAGACTAGTGATT, Forward miR-135a-5p CCGCGGCTATGGCTTTTTATTCC, Forward miR-138-5p CGGGC- AGCTGGTGTTGTGAATC, Forward U6 GCGGTCGCAAGGATGACACG, Universal reverse CCAGTGCAGGGTCCGAGGTAT.

### 2.3. Protein Extraction, Western Blot (WB), and Band Quantification

Proteins were isolated from malignant and corresponding non-malignant thyroid tissues. Approximately 100 mg was resuspended in 1 mL of cold extraction buffer of pH 8.0 (20 Mm Tris HCl, 137 Mm NaCl, 10% glycerol, 1% NP-40, 2 Mm EDTA) with Protease Inhibitor Cocktail (p8340; Sigma-Aldrich, St. Louis, MO, USA) and phosphatase inhibitors (p5726, Sigma-Aldrich, St. Louis, MO, USA). The samples were then homogenized using an LT QIAGEN homogenizer (QIAGEN, Hilden, Germany) at 50 Hz for 10 min. In the next step, samples were centrifuged for 20 min at 12,000 rpm at 4 °C as to remove debris. After centrifugation, the supernatant was aliquoted to be used for the determination of protein concentration with a Bicinchoninic acid assay BCA protein assay kit (Pierce, Rockford, IL, USA) and Western blot. For Western blot, a total of 100 µg protein was loaded for each sample. Sixty-eight malignant and fourteen non-malignant isolates were separated on 7.5% polyacrylamide gel and transferred onto a 0.45 µm nitrocellulose membrane (Amersham Protran, GE Healthcare Life Science, Marlborough, MA, USA). After transfer, membranes were blocked by 5% milk in Tris-buffered Saline with 0.1% Tween 20 for one hour at room temperature and then incubated with anti-phosphotyrosine (Tyr397) FAK (clone 31H5L17, Cat# 700255, RRID: AB_2532307; dilution 1:500) and anti-β-actin (clone AC-15, Cat# MA1-91399, RRID: AB_2273656, ThermoFisher Scientific, Waltham, MA, USA; dilution 1:1000) antibodies overnight at 4 °C. Secondary antibodies were incubated for 30 min at room temperature at a dilution of 1:2000 in Tris buffer saline with 1% milk. The signal was enhanced by a vectastain ABC kit (Vector Laboratories, Newark, CA, USA). After each incubation, membranes were washed with Tris-buffered saline (0.05 M Tris with 0.15 M NaCl, pH 7.6) The bands were visualized on a photographic film (MXBE film 13 × 18, Carestream Health, Inc., Rocheste, NY, USA) after incubation with an ECL (enhanced chemiluminescence substrate, ThermoFisher Scientific, Waltham, MA, USA). Protein bands were quantitated by densitometry using TotalLab v2.01 software (Nonlinear Dynamics, New Castle, UK) and normalized to β-actin and an interblot control (protein extract loaded in each blot as a control for interblot variability).

### 2.4. Immunohistochemistry and Semiquantitative Scoring Method

Immunohistochemical staining was conducted as reported previously [26], using the same antibody as for Western blot (clone 31H5L17 cat no 700255 RRID: AB2532307, dilution 1:200). In short, slides were deparaffinized in xylene and rehydrated in graded ethanol baths. Endogenous peroxidases were blocked by adding hydrogen peroxide. Unspecific binding was blocked by incubation with normal horse serum for 20 min at room temperature. Primary antibodies were incubated overnight at 4 °C while secondary antibodies were kept for 35 min at room temperature. The signal was enhanced by a vectastain ABC kit (cat no PK-6100, Vecor Laboratories, Newark, CA, USA). The reaction was visualized by a 3, 3′-diaminobenzidine peroxidase substrate kit, DAB (cat no SK-4100, Vecor Laboratories, Newark, CA, USA). In addition, a negative control was performed by omitting primary antibodies, and no staining was observed. Two independent researchers, blinded to clinical data, were evaluating the immunoreactivity of samples using an Axio Imager 1.0 microscope (Carl Zeiss, Jena, Germany) with a Canon A640 Digital Camera System. The final score of each sample was calculated by multiplying the proportion of immunoreactive cells with their corresponding staining intensity, as described in [27]. The intensity grading was as follows: 0, absence of staining; 1, weak staining; 2, moderate staining; 3, intense staining. The formula for the score calculation was as follows: score = 0 × (ratio of not stained) + 1 × (ratio of weakly stained) + 2 × (ratio of moderately stained) + 3 × (ratio of intensively stained cells). The final grades were determined using the following score ranges: grade 0: score 0; grade 1: scores 0–1; grade 2: scores 1–2; grade 3: scores 2–3. Since the presence of pY397-FAK was observed in different cellular compartments, the scoring results were evaluated separately for membrane (M), cytoplasm (C), and nucleus (N).

### 2.5. Statistical Analysis

Differences in the expression of three miRs and pY397-FAK between malignant and non-malignant tissue were tested using Student’s *t*-test for paired samples. Spearman’s and Pearson correlations were used for the association of IHC stainings, miRs’ and pY397-FAK expression (densitometry quantification), and diagnosis, graded according to dedifferentiation level. In the aim to test differences in expression of each miR and pY397-FAK (immunohistochemical scores) between a follicular and classical variant of PTC, we used Student’s and Chi-square tests, respectively. Finally, for the association of miRs’ expression with clinicopathological parameters we used Student’s *t*-test and Pearson correlation. Statistical analysis was performed on SPSS 26 (SPSS, Chicago, IL, USA), and a *p*-value < 0.05 was considered statistically significant.

## 3. Results

### 3.1. Relative Expression of miR-7-5p, miR-135a-5p, miR-138-5p, and pY397-FAK in Healthy and Malignant Thyroid Tissue

**Figure 1:**
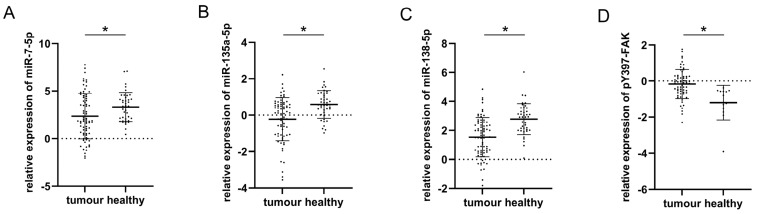
Comparison of plotted values of RT-qPCR analysis of miRs-7-5p (**A**), -135a-5p (**B**), and -138-5p (**C**); plotted values of densitometry quantification of pY397-FAK Western blot normalized to β-actin (**D**) in malignant and healthy tissue. Mean values and standard deviations are represented with horizontal lines. All data were transformed logarithmically. * *p* < 0.05.

The plotted values of qPCR analysis for three analyzed miRs in malignant and healthy thyroid tissue are presented in Figure 1A–C. All three miRs were upregulated in conditionally healthy tissue compared to their malignant counterparts, which was confirmed by paired Student’s *t*-test. The respective mean values and standard deviations along with *t*-test *p*-values were as follows: miR-7-5p (2.57 ± 2.45 in tumor vs. 3.32 ± 1.52 in healthy, *p* = 0.032), miR-135a-5p (−0.93 ± 1.04 in tumor vs. 0.58 ± 0.76 in healthy, *p* = 0.000), miR-138-5p (1.75 ± 1.46 in tumor vs. 2.78 ± 1.06 in healthy, *p* = 0.000). Figure 1D represents the results of the densitometric evaluation of pY397-FAK Western blot results. It shows an inverse trend compared to miRs as its expression is significantly elevated in tumor tissue with respect to healthy (0.145 ± 0.64 vs. −1.23 ± 1, *p* = 0.003).

### 3.2. Correlation between pY397-FAK IHC Scores (Membranous, Cytoplasmic, and Nuclear); pY397-FAK Western Blot Analysis, miRs (-7-5p, -135a-5p, -138-5p) qPCR Expression, and Diagnosis Graded According to Dedifferentiation Level and Aggressiveness

In order to evaluate the association of pY397-FAK and its abovementioned regulators with histotype aggressiveness (and level of histologic dedifferentiation), we performed Spearman’s nonparametric and Pearson’s parametric correlation tests where appropriate. Table 1 presents a matrix of correlation coefficients and their statistical significance between all analyzed parameters. The analysis shows that membranous and cytoplasmic pY397-FAK staining (but not nuclear), as well as overall immunoexpression detected by Western blot, show a trend of gradual overexpression with increasing histotype aggressiveness. Of all miRs, only miR-135a-5p demonstrated a significant negative correlation with sorted diagnostic categories, i.e., exhibiting a considerable drop with increasing dedifferentiation. Furthermore, all miRs were positively correlated with each other, while pY397-FAK IHC analysis of individual compartments showed a link between membranous and cytoplasmic, but not nuclear, expression. It is noteworthy that no association was found between the selected miRs and pY397-FAK WB or IHC analysis.

### 3.3. Correlation between miRs (-7-5p, -135a-5p, -138-5p) Expressions and Clinicopathological Factors of PTC

Correlations between the selected miRs and adverse clinicopathological findings are presented in Table 2. Among the observed traits we considered were age, gender, tumor size, intraglandular dissemination, presence of lymph node metastasis, degree of tumor infiltration, extrathyroid invasion, pT, and pTNM stage. The analysis did not show significant differences between miR distribution in any comparison.

### 3.4. The Performances of miRs (-7-5p, -135a-5p, -138-5p) Expressions and pY397-FAK IHC Scores (Membranous, Cytoplasmic, and Nuclear) as Discriminators between Follicular and Classical Variants of PTC

**Figure 2:**
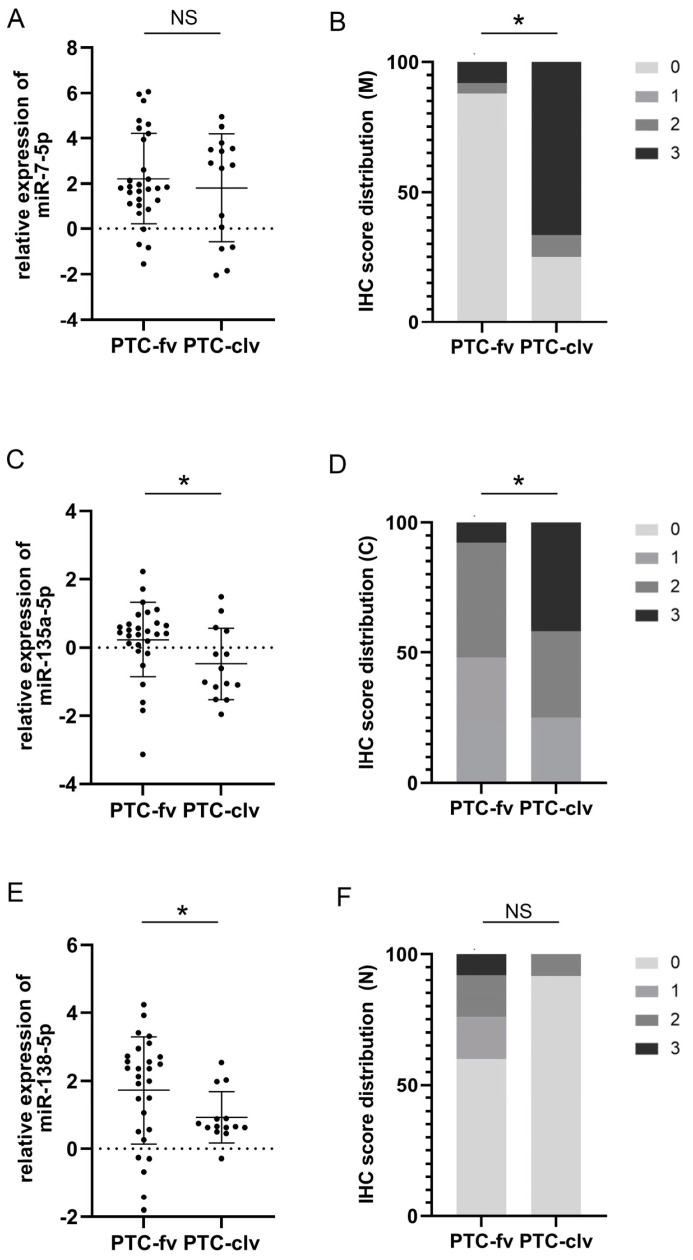
Comparison of plotted values of RT-qPCR analysis of miRs-7-5p (**A**), -135a-5p (**C**), and -138-5p (**E**) in PTC-fv and PTC-clv. Mean values and standard deviations are represented with horizontal lines. All data were transformed logarithmically. Distribution of immunohistochemical scores of pY397-FAK in the membranous compartment (**B**); cytoplasmic compartment (**D**) and nuclear compartment (**F**) between PTC-fv and PTC-clv. * *p* < 0.05. NS stands for non-significant.

**Figure 3:**
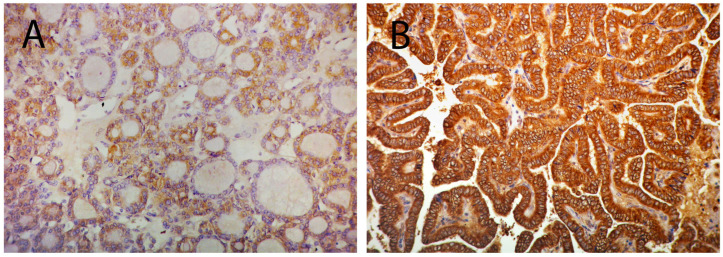
Micrograph illustrating low expression of pY397-FAK in the membrane, cytoplasm, and nucleus PTC-fv (**A**); and strong expression of pY397-FAK in all subcellular compartments in PTC-clv (**B**). Original magnification 20×.

Further on, we attempted to assess the usefulness of suggested markers as discriminators between the two most frequent forms of PTC: follicular and classical variants. Figure 2A,C,E shows the differences in the relative expression of each examined miR between follicular and classical variants. *t*-test statistics demonstrated significant differences in the expression of miR-135a-5p (0.3 ± 1.05 for fv; −0.48 ± 1.05 for clv; *p* = 0.029) and miR138-5p (1.72 ± 1.58 for fv; 0.92 ± 0.75 for clv; *p* = 0.033), but not miR-7-5p (2.21 ± 2 for fv; 1.81 ± 2.38 for clv; *p* = 0.557) between these histotypes. The right side of Figure 2B,D,F represents charted immunohistochemical scores of pY397-FAK in both PTC subtypes. Differences in distribution were determined by χ^2^ statistics. Analysis shows differences in pY397-FAK expression only when considering membranous (*p* = 0.000) and cytoplasmic (*p* = 0.046) compartments. In both cases, the follicular variant tended to be more immunonegative than the classical variant. When considering membranous expression, only 0.08% of follicular variant cases received the highest IHC score, as opposed to 66.7% of cases of the classical variant (*p* = 0.000). Cytoplasmic distribution showed a similar tendency with 0.08% of highly scored cases in the follicular and 41.7% in the classical variants (*p* = 0.046). Figure 3 shows representative cases of pY397-FAK immunostaining in PTC-fv (3A) and PTC-clv (3B). The follicular variant displayed weak diffuse positivity of pY397-FAK in the membrane, cytoplasm, and nucleus, with occasional unstained follicles. The classical variant shows strong expression of pY397-FAK in all subcellular compartments on the entire tumor section with sporadic immunonegative nuclei.

## 4. Discussion

MiRs-7, -135a, and -138 are transcriptional regulators recognized for maintaining cellular homeostasis through buffering gene expression and stabilizing molecular networks. Their deregulation leads to disturbances in the global transcriptional machinery of the cell, possibly driving several pathological processes including cancer. One of their targets, FAK, is on the intersection of numerous signaling transduction cascades regulating various aspects of fundamental cell behavior, including, but not limited to, motility, communication, and response to environmental cues [28,29]. Therefore, it is reasonable to assume that derangements of miR expression influence the activity of FAK among other targets, thereby altering cell behavior, morphology, and phenotype.

PTC is a well-differentiated malignancy usually appearing in two conventional forms: follicular as a more indolent subtype; and the classical variant with more deteriorating consequences. However, in about 5% of PTC cases, dedifferentiation occurs, giving rise to rare, unusual histotypes exhibiting increasingly aggressive traits [30,31]. Given the rarity of these forms, they are poorly described in the literature and their biological and clinical properties are largely unknown. At the end of the dedifferentiation process, ATC arises as an undifferentiated histotype exhibiting stem-like properties with high proliferative potential. Although some ATC may arise de novo, an increasing amount of evidence also suggests that accumulation of genetic alterations drives the dedifferentiation of PTC towards ATC [32,33]. Some of this evidence includes frequent histopathologic coexistence of PTC components in ATC lesions and a history of PTC in most ATC cases.

In the first part of this paper, by examining the expression of pY397-FAK and three of its known miR regulators in conditionally healthy tissue and a palette of increasingly dedifferentiated thyroid neoplasia, we aimed to gain insight into the process of continuous malignancy progression towards undifferentiated histotypes.

Although several exceptions are noted, the literature data indicate that malignantly transformed tissue is accompanied by lower levels of miR-7, miR-135a, and miR-138 compared to their healthy counterparts, which is strongly corroborated by our findings in thyroid cancer [17,18,19]. All three miRs were downregulated in malignant compared to adjacent healthy tissue. However, pY397-FAK demonstrated an opposing trend, showing an increase in carcinoma tissue. These findings are in accordance with several lines of evidence that demonstrated direct targeting and downregulation of FAK by the mentioned miRs. Although these results were acquired on postoperative removed tissue, they might also have potential as preoperative markers in the early diagnostics of thyroid nodules. A challenge in thyroid diagnostics is obtaining information regarding malignant potential on preoperative samples so as to avoid unnecessary surgery. Therefore, further studies are needed to assess their capacity on preoperative samples obtained by fine needle aspiration biopsy.

Further on, we investigated whether the trend of decline in miR levels and increase in pY397-FAK remains throughout the series of dedifferentiation events from PTC-fv to ATC. Given its compartment-specific role, the expression of pY397-FAK was separately evaluated and correlated in the nuclear, cytosol, and membrane compartment by IHC, and its total expression was also analyzed in a crude tissue extract by Western blot. Our results indicate a significant rise of pY397-FAK in the membranous and cytoplasmic compartments with increasing dedifferentiation. Much to our surprise, the correlation of the nuclear compartment was lacking. This possibly indicates a more decisive role of FAK in signal integration, locomotion, and adherence during malignant transformation, rather than its nuclear function in transcription factor regulation. It is also noteworthy that cytoplasmic and membranous pY397-FAK were strongly mutually correlated; however, membranous and nuclear staining showed a reverse correlation, possibly suggesting a translocation process from the nucleus to the cytoplasm. Finally, total pY397-FAK measured by Western blot also showed an increase accompanying gradual dedifferentiation, once again implying a dominant membranous and cytoplasmic role of pY397-FAK in comparison to nuclear, given that the minor part of pY397-FAK is localized in the nucleus.

Contrary to our expectations, no miR correlated with pY397-FAK. A probable explanation would be that the transcriptional regulation of FAK by miRs is not synchronized with its posttranslational phosphorylation. Further research evaluating FAK unmodified by phosphorylation as a missing link between miRs and pY397-FAK would shed more light on its transcriptional and posttranslational regulation.

Among miRs, only miR-135a-5p exhibited a significant decrease with dedifferentiation increment. Our sample series regrettably also did not show an association between miRs’ expression and the clinicopathological data of PTC including the presence of lymph node metastasis, intraglandular dissemination, degree of tumor infiltration, extrathyroid invasion, or disease stage. However, all three miRs were strongly mutually correlated, demonstrating a concomitant expression pattern and probably a common regulatory mechanism.

In all, we suggest that alterations in the expression of miR-135a-5p and membranous and cytoplasmic pY397-FAK during thyroid malignant transformation can qualitatively modify malignant cells towards more aggressive phenotypes and facilitate PTC to dedifferentiate into ATC via disruption of the mentioned regulatory pathways. Nevertheless, when it comes to the development of unfavorable clinicopathological features of PTC, miRs do not seem to have an important effect. In our previous research, we demonstrated correlations between pY397-FAK in all analyzed compartments with multiple unfavorable characteristics of PTC; however, miRs do not seem to follow this trend.

Given the promising results regarding the gradual change of biomarker expression between examined histologic categories with increasing dedifferentiation levels, the second part of our study was dedicated to examining whether the chosen markers could be beneficial in discriminating PTC-fv from PTC-clv. Namely, although these histotypes are defined by disparate tissue architecture and cytological characteristics, their clinical equivalency was historically assumed up until recently. Nowadays we know they represent unique clinical entities with different long-term outcomes; however, their repeated overlapping of ultrasonographical and pathohistological features makes their differential diagnosis difficult. Moreover, an encapsulated form of PTC-fv is gaining acceptance among the clinical community as a benign lesion requiring more conservative surgery and lighter postoperative treatment. Even a reclassification of this subtype to Noninvasive Follicular Thyroid Neoplasm with Papillary-like Nuclear Features (NIFTP) is proposed reflecting its indolent behavior [34]. On the other hand, a recent large retrospective study encompassing 1293 patients concluded that compared to PTC-fv, PTC-clv was associated with multiple features of high-risk disease [35]. Since the main challenge in the clinical management of thyroid neoplasia is preventing over-treatment of patients with low-risk disease while promptly identifying high-risk patients in need of a harsher treatment, a molecular marker capable of differentiating these histotypes could facilitate clinical decision-making regarding the extent of surgical procedure and postoperative treatment.

According to our findings, miRs-135a-5p and -138-5p, but not miR-7-5p were competent discriminators between PTC-fv and PTC-clv, demonstrating significantly lower expression in the more aggressive classic histotype, as expected by previous findings. Regarding IHC findings, membranous and cytoplasmic expression of pY397-FAK was evidently lower in PTC-fv than PTC-clv, while nuclear staining showed no significant differences in distribution. Therefore, we conclude that miRs-135a-5p and -138-5p and the evaluation of pY397-FAK in the membrane and cytoplasmic compartment could be a new promising potent tool for guiding thyroid cancer management.

## 5. Conclusions

To conclude, although all three miRs were upregulated in healthy compared to malignant tissue, only miR-135a-5p followed the downregulation trend through dedifferentiation all the way to ATC. As for pY397-FAK, its increase in expression in the membrane and cytoplasm accompanies the same process. Diagnostically, miRs-135a-5p and -138-5p as well as membranous and cytoplasmic pY397-FAK had the capacity to differentiate between PTC-clv and PTC-fv. Further research is needed to fill the gap in our understanding of the connection between selected miRs and pY397-FAK.

## Figures and Tables

**Table 1:** Correlation between: pY397-FAK IHC staining; miRs-7-5p, 135a-5p, and 138-5p expressions; pY397-FAK WB score and thyroid histotype aggressiveness.

	Correlation Parameters	IHC(M) pY397-FAK	IHC(C) pY397-FAK	IHC(N) pY397-FAK	qPCR miR-7-5p	qPCR miR-135a-5p	qPCR miR-138-5p	WB pY397-FAK	Histotype Agg.
**IHC(M)** **pY397-FAK**	r		0.755	−0.374	−0.031	−0.108	−0.155	0.495	0.367
	*p*		**0.000 ^a^**	**0.002 ^a^**	0.810 ^a^	0.416 ^a^	0.237 ^a^	**0.000 ^a^**	**0.002 ^a^**
**IHC(C)** **pY397-FAK**	r	0.755		−0.214	0.052	−0.033	0.001	0.470	0.258
	*p*	**0.000 ^a^**		0.080 ^a^	0.690 ^a^	0.804 ^a^	0.993 ^a^	**0.000 ^a^**	**0.034 ^a^**
**IHC(N)** **pY397-FAK**	r	0.374	−0.214		0.030	0.037	0.046	−0.112	−0.180
	*p*	**0.002 ^a^**	0.080 ^a^		0.817 ^a^	0.782 ^a^	0.730 ^a^	0.415 ^a^	0.142 ^a^
**qPCR** **miR-7-5p**	r	−0.031	0.052	0.030		0.317	0.597	−0.100	0.042
	*p*	0.810 ^a^	0.690 ^a^	0.817 ^a^		**0.008 ^b^**	**0.000 ^b^**	0.460 ^b^	0.723 ^a^
**qPCR** **miR-135a-5p**	r	−0.108	−0.033	0.037	0.317		0.451	−0.097	−0.308
	*p*	0.416 ^a^	0.804 ^a^	0.782 ^a^	**0.008 ^b^**		**0.000 ^b^**	0.480 ^b^	**0.008 ^a^**
**qPCR** **miR-138-5p**	r	−0.155	0.001	0.046	0.597	0.451		−0.116	−0.097
	*p*	0.237 ^a^	0.993 ^a^	0.730 ^a^	**0.000 ^b^**	**0.000 ^b^**		0.394 ^b^	0.416 ^a^
**WB** **pY397-FAK**	r	0.495	0.470	−0.112	−0.100	−0.097	−0.116		0.255
	*p*	**0.000 ^a^**	**0.000 ^a^**	0.415 ^a^	0.460 ^b^	0.480 ^b^	0.394 ^b^		**0.043 ^a^**
**Histotype agg.**	r	0.367	0.258	−0.180	0.042	−0.308	−0.097	0.255	
	*p*	**0.002 ^a^**	**0.034 ^a^**	0.142 ^a^	0.723 ^a^	**0.008 ^a^**	0.416 ^a^	**0.043 ^a^**	

IHC: immunohistochemical staining; (M): membranous staining; (C): cytoplasmic staining; (N): nuclear staining. qPCR: quantitative PCR; WB: Western blot analysis. Histotype agg.: Histotype aggressiveness, scored as detailed in the Materials and Methods section. r-correlation coefficient, *p*-value of statistical significance. *p* < 0.05 was considered statistically significant. Statistically significant results are bolded. Correlations: ^a^ Spearman’s or ^b^ Pearson’s.

**Table 2:** Association of miR-7-5p, miR-135a-5p, and miR-138-5p expressions with clinicopathological factors of PTC.

		N (75)	miR-7-5p	*p*	miR-135a-5p	*p*	miR-138-5p	*p*
**Age in Years (Range)**		14–89	r = −0.078	0.505	r = −0.122	0.618 ^a^	r = 0.059	0.618 ^a^
**Gender**	Male	16	3.385 ± 2.13	0.059 ^b^	−0.103 ± 1.333	0.68 ^b^	1.867 ± 1.11	0.287 ^b^
	Female	60	2.117 ± 2.403		−0.25 ± 1.16		1.446 ± 1.406	
**Tumor size (mm)**	range	2–120	r = −0.130	0.265 ^a^	r = −0.067	0.578 ^a^	r = −0.025	0.831 ^a^
**Intraglandular dissemination**	Absent	41	2.711 ± 2.456	0.2 ^b^	−0.2 ± 1.275	0.864 ^b^	1.426 ± 1.608	0.48 ^b^
	Present	34	1.998 ± 2.287		−0.248 ± 1.092		1.653 ± 0.997	
**Lymph node** **metastasis**	Absent	62	2.334 ± 2.327	0.678 ^b^	−0.148 ± 1.108	0.221 ^b^	1.551 ± 1.363	0.78 ^b^
	Present	13	2.64 ± 2.768		−0.627 ± 1.56		1.431 ± 1.363	
**Degree of tumor infiltration**	1/2	40	2.604 ± 2.236	0.3 ^b^	−0.021 ± 1.079	0.5 ^b^	1.744 ± 1.501	0.134 ^b^
	3/4	32	2.001 ± 2.587		−0.577 ± 1.246		1.255 ± 1.116	
**Extra-thyroid** **invasion**	Absent	54	2.41 ± 2.464	0.902 ^b^	−0.211 ± 1.224	0.905 ^b^	1.54 ± 1.422	0.933 ^b^
	Present	21	2.332 ± 2.253		−0.249 ± 1.115		1.51 ± 1.187	
**pT**	Low (1/2)	54	2.409 ± 2.408	0.92 ^b^	−0.224 ± 1.19	0.981 ^b^	1.601 ± 1.461	0.579 ^b^
	High (3/4)	21	2.351 ± 2.408		−0.217 ± 1.204		1.419 ± 1.178	
**pTNM**	Low (1/2)	52	2.422 ± 2.317	0.648 ^b^	−0.143 ± 1.168	0.323 ^b^	1.566 ± 1.385	0.525 ^b^
	High (3/4)	22	2.144 ± 2.539		−0.452 ± 1.244		1.347 ± 1.224	

N: number of cases; *p*: value of statistical significance. *p* < 0.05 was considered statistically significant; r-correlation coefficient; pT: pT status (as detailed in the Materials and Methods section); pTNM: pTNM stage (as detailed in the Materials and Methods section); ^a^ Pearson’s correlation; ^b^ Student’s *t* test.

## Data Availability

The data presented in this study are available on request from the corresponding author.
